# Testing evolutionary conflict theories for sexual and physical intimate partner violence in Sub-Saharan Africa

**DOI:** 10.1017/ehs.2022.58

**Published:** 2022-12-19

**Authors:** Janet A. Howard, Mhairi A. Gibson

**Affiliations:** Department of Anthropology and Archaeology, University of Bristol, 43 Woodland Road, Bristol BS8 1UU, UK

**Keywords:** Sexual conflict, paternity certainty, domestic violence, evolutionary anthropology

## Abstract

Intimate partner violence (IPV) refers to physical, sexual and psychological violence. Here an evolutionary approach is used to compare risk factors for male-to-female IPV perpetration, analysing physical and sexual IPV separately. Two hypotheses based on sexual conflict theory have been applied to IPV perpetration, but they remain largely untested using empirical data: (a) men perpetrate IPV in response to a perceived threat to their paternity certainty; and (b) IPV is caused by men pursuing a higher fertility optima than their partners, either within marriage (reproductive coercion) or outside marriage (paternal disinvestment). Demographic Health Survey data from couples in 12 sub-Saharan African countries (*n* = 25,577) were used to test these evolutionary hypotheses, using multilevel models and controlling for potential social and environmental confounds. The results show that evolutionary theory provides important insight into different risk factors by IPV type. Indicators of paternity concern are associated with an increased risk of both physical and sexual IPV, indicators of paternal disinvestment are associated with an increased risk of physical IPV only, while reproductive coercion is not associated with either IPV type. The risk factors identified here correspond with proximate-level explanations for IPV perpetration, but an evolutionary interpretation explains why these particular factors may motivate IPV in certain contexts.

## Graphical abstract

**Figure:**
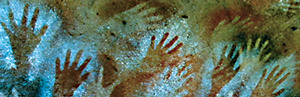

**Social media summary:** Evolutionary approach offers explanations for risks factors for committing physical and sexual IPV in sub-Saharan Africa

## 1. Introduction

Intimate partner violence (IPV) is defined as physical, sexual and emotional abuse as well as controlling behaviours towards an intimate partner (WHO, 2012). Prevalence varies by country, but it is estimated that between 15 and 71% of women experience physical IPV and 10–50% experience sexual IPV in their lifetimes (Garcia-Moreno et al., 2006, 2012). Intimate partner violence is a priority for policymakers owing to the significant detrimental impact on women and their children's health and wellbeing (UN, 2016). Understanding why men perpetrate IPV is a step towards designing initiatives aimed at behavioural change.

Multiple theories have been put forward to try to explain why men perpetrate physical and sexual IPV. The focus of this paper is to examine how certain evolutionary theories can be applied to explore motivations for male-to-female IPV; however, it is important to recognise the contributions of non-evolutionary social science theories which are also applied to understand IPV behaviour. These include: feminist theory which attributes IPV to a male desire to control women (Dobash and Dobash, 1979; García-Moreno et al., 2005); family violence theory which anticipates sex-symmetry in perpetration (Straus & Gelles, 1986; Dutton & Nicholls, 2005); relative resource theory, which predicts that women with more resources than their partners are at greater risk of IPV (Allen & Straus, 1975; Atkinson et al., 2005); and social learning theory, which proposes that witnessing or experiencing IPV in childhood predicts IPV perpetration in adulthood (O'Leary, 1988). The current prevailing theory endorsed by the WHO uses an ecological framework and proposes that factors at the individual, couple, community and national levels interact and contribute towards IPV occurrence (Heise, 1998; WHO/LSHTM, 2010). It is notable that these non-evolutionary frameworks rarely distinguish between different types of IPV.

Non-evolutionary theories of IPV can provide a proximate level understanding of the immediate causes of behaviour, but evolutionary theories uniquely engage with ultimate explanations, by also considering how behavioural traits evolved (Tinbergen, 1963). Proximate and ultimate levels of explanation are complementary and are incorporated into evolutionary theories, which anticipate that individuals will behave in a way that enhances their evolutionary fitness. Applying this to IPV, it is predicted that men will perpetrate IPV in contexts where they gain an evolutionary fitness benefit from doing so (Krebs & Davies, 2009), and that IPV may be a facultative response by men to their local ecology (Scelza et al., 2020). Past work in this area has been controversial, in part owing to analysis of rape in relation to evolutionary theory (see Vandermassen, 2011 for a review of the controversies). Here it should be emphasised that evolutionary fitness is defined as an individual's contribution to the gene pool of future generations, often measured by actual, or proxies for, an individual's reproductive success (Nettle et al., 2013). More specifically, sexual conflict theory is often applied to explain IPV. This proposes that conflict in evolutionary interests between the sexes arises when the optimal value of a trait (e.g. parental investment or number of offspring) differs for each sex, resulting in the evolution of strategies and counter strategies by males and females to achieve their own reproductive goals (Parker, 2006). Sexual conflict theory was developed in animal studies, and the applicability of these principles to relationships between the sexes in humans remains uncertain, particularly in monogamous societies where reproductive costs are shared (Borgerhoff Mulder & Rauch, 2009; Moya et al., 2016; Lawson et al., 2021). However, two potential sources of sexual conflict have been put forward to explain male-to-female IPV which are brought together in this paper: paternity uncertainty and reproductive conflict.

Paternity uncertainty describes men's concern about their partners pursuing extra-pair sex and their own risk of non-paternity (Buss, 1996). It is suggested that men have evolved strategies to prevent this, including morphological adaptations (e.g. sperm competition; Pham & Shackelford, 2014) and behavioural adaptations such as mate-guarding and IPV (Hartung, 1985; Goetz et al., 2008). It has been argued that male sexual jealousy is a universal cognitive function selected to address the issue of female infidelity and paternity uncertainty (Goetz et al., 2008). Studies carried out in the USA found that men who perpetrated sexual IPV were more likely to have accused their partner of sexual infidelity, e.g. (Goetz & Shackelford, 2009), or to report suspicion that their partner was having extra-pair sex (McKibbin et al., 2011). Physical IPV has also been found to be associated with accusations of female sexual infidelity and a higher perceived risk of partner infidelity (Kaighobadi et al., 2009). Supporting these findings, some non-evolutionary studies conducted in low- to middle-income countries have identified suspicion of partner infidelity as a key risk factor for violence (e.g.Townsend et al., 2011).

Reproductive conflict, as applied to IPV, describes the proposal that men have a higher optimum fertility than women. This proposal is based on the idea that females carry a higher minimum cost of reproductive investment compared with males (in humans, through gestation and lactation). Given this higher level of female investment, larger numbers of offspring may be more costly for female survival, while males may be able to move on to another female to continue reproduction if their mate dies (but see Moya & Snopkowski 2016 for discussion). The evidence in humans is circumstantial, e.g. surveys documenting men's preference for larger family sizes than women (Mason & Smith, 2000); however, two scenarios based on conflict over fertility between men and women have been put forward as explanations for IPV. Firstly, reproductive coercion suggests that men use IPV (physical and sexual) to achieve higher fertility goals within their relationship through pregnancy coercion, birth control sabotage or influencing the outcome of a pregnancy (Miller et al., 2010). Indirect support comes from studies which found that men who perpetrated IPV had higher odds of fathering a pregnancy (Christofides et al., 2014) and fathering three or more children (Raj et al., 2006), that men who perpetrated physical IPV gained more frequent sexual access to their partner (Barbaro & Shackelford, 2016), and that men with higher fertility preferences than their wives used IPV to oppose their wives’ contraceptive use (Forrest et al., 2018). Secondly, paternal disinvestment suggests that IPV (primarily physical) is triggered when men divert their resources (in the broadest sense) away from their partner to achieve higher fertility outside the relationship, for example through extra-pair sex, polygamy or changing relationships (Stieglitz et al., 2011). Access to resources and conflict over male infidelity are known risk factors for IPV (Abrahams et al., 2006). Higher risk of IPV in polygamous marriages has been found in several studies, e.g. in Kenya (Makayoto et al., 2013; Kimuna & Djamba, 2008), and qualitative studies have also described how disagreements over finances and extramarital relations precipitate IPV (Fuller, 2001; Sedziafa et al., 2018).

While this literature provides some evidence that both physical and sexual IPV may be motivated by evolutionary fitness concerns, it is not conclusive. Pair bonding appears to be a successful strategy in humans in many contexts. Men benefit from access to mating, which may be less costly than seeking alternative new mates, and the ability to invest in and protect their offspring (Quinlan, 2008). The potential fitness costs of perpetrating IPV and jeopardising this pair bond include retaliation from relatives or exclusion from social networks (Clark et al., 2010), a negative impact on their partner's fertility, and therefore their own reproductive success, owing to the health consequences associated with IPV (Hill et al., 2016), and risk of their wife's defection from the relationship (Marlowe, 2000). Therefore, it is questionable whether IPV is an effective fitness enhancing strategy. However, IPV is also a widespread human behaviour that is perpetrated at high levels in many societies, and requires an explanation (WHO, 2012).

Physical and sexual IPV are often grouped together in theory and analysis; however, several differences suggest that they merit separate investigation. Physical IPV refers to a range of generic violent acts of differing severity which could take place between any two individuals. Sexual IPV is distinguished by its sexual nature and typically refers to a narrower range of behaviours, usually forced sexual acts or forced intercourse (Basile et al., 2014). Sexual IPV can also result in conception which may be relevant from an evolutionary perspective. Few studies have analysed sexual and physical IPV separately, and it is unclear whether they are on a continuum of behaviour, or whether perpetrator motivations differ (Krebs et al., 2011). The available evidence suggests that fewer, rather than different, variables are significantly associated with sexual IPV (Townsend et al., 2011; Fulu et al., 2013). Physical IPV has been associated with men's poverty, lower education, involvement in violence with other men, drug and alcohol abuse, community norms, poor mental health and older age, but these variables were not associated with sexual IPV in the same populations (Abrahams et al., 2004, 2006; Koenig et al., 2006; Sambisa et al., 2010; Fulu et al., 2013). It has also been reported that sexual IPV has more risk factors in common with non-partner sexual violence than with physical IPV (e.g. Fulu et al., 2013; Jewkes et al., 2013).

Here, we test two hypotheses, examining physical and sexual IPV separately. First, we test the hypothesis that men exposed to indicators of paternity concern will be more likely to commit IPV. This is explored using multiple individual-level and community-level proxies for paternity concern. Second, we test the hypothesis that men whose reproductive interests conflict with those of their wives will be more likely to commit IPV. Here, we predict that sexual IPV will be more strongly associated with variables indicative of reproductive coercion, and physical IPV will be more strongly associated with variables indicative of paternal disinvestment.

## 2. Methods

Demographic Health Survey (DHS) datasets from countries in sub-Saharan Africa were used, which survey around 3,000 men and 10,000 women in each country. The sample was limited to one region to reduce variation resulting from geographical and cultural factors. An optional IPV module was used by 18 surveys undertaken since 2017, of which 12 were suitable for use (IPV data for husbands and wives could not be matched in the other six datasets). DHS datasets provide large sample sizes of highly relevant data; however, as the DHS data were not collected for these specific research questions, proxy indicators for relevant behaviours are used. Men and women who had been married/cohabiting for a year or more were included in the sample, with polygamous men included once in a couple with their first wife. Respondents whose ethnic group was unknown or from an ethnic group with fewer than 50 members were excluded. This provided a final sample of 25,577 couples from 103 identifiable ethnic groups, in 12 countries (Burkina Faso, Chad, Ethiopia, Gambia, Ghana, Ivory Coast, Kenya, Malawi, Mali, Nigeria, Togo and Zambia).

The outcome variable in both hypotheses was women's experience of physical and sexual IPV in the 12 months preceding the survey. This was time restricted (rather than using lifetime experience) to match men's current circumstances with their recent IPV perpetration as far as possible. The IPV perpetration data is not collected from men, but men who perpetrated IPV were identified by matching husbands and wives from the male and female datasets. Evidence showing alignment between women's reported experience of IPV and their husbands’ reports of IPV perpetration supports this approach (Halim et al., 2018; Jewkes et al., 2017; Hoffman et al., 1994; Barker et al., 2015). The DHS asks women whether they have experienced any physical IPV (being pushed, shaken, slapped, punched, kicked, dragged, beaten up, choked or attacked with a weapon) or sexual IPV (being forced, physically or in any other way, to have sexual intercourse, or perform sexual acts). Women who reported they had experienced one or more of these behaviours, either sometimes or often, were coded as having experienced IPV.

The statistical models controlled for variables which have been shown to be associated with IPV in other studies. Many variables have been shown to be associated with IPV in the literature (see Supplementary Table S5 for full references). All relevant variables available in the DHS dataset were used as control variables in this analysis. This included household wealth (quintiles as coded by the DHS), household location (urban or rural), husband's education (none, primary, secondary or higher), husband's religion (Muslim, Christian, other/none), husband's alcohol use (yes or no), husband's age, wife's age and childhood exposure to physical violence (yes or no). To control for the husband's gender attitudes, the models also included the husband's engagement in transactional sex (yes or no) and how many IPV justifications he agreed with (0–5): if his wife goes out without telling him, neglects the children, argues with him, refuses to have sex with him, or burns the food. Childhood exposure to physical violence was calculated using women's responses to the question of whether their father beat their mother at the ethnic group level, as men's individual responses were not captured. Experience/perpetration of physical IPV was controlled for in the models examining sexual IPV, and sexual IPV experience/perpetration was controlled for in the models examining physical IPV. The paternity concern models also controlled for the number of marriages (one or more than one), as this would be confounded with the respondent's sexual history.

Different independent variables were used to test each hypothesis. For hypothesis 1, that men exposed to indicators of paternity concern will be more likely to commit IPV, several proxy indicators of paternity concern were tested in the models. The DHS asks women three questions (not time bound) relevant to paternity concern: whether her husband is jealous when she talks to other men; whether he accuses her of being unfaithful; and whether he insists on knowing where she is at all times. Other indicators used were whether the husband and wife had sex before marriage, which may be indicative of attitudinal differences (yes or no), and their lifetime number of sexual partners (treated as a categorical variable, 1, 2, 3 and 4+, as the data was highly skewed). Indicators of men and women's sexual activity in the ethnic group (the percentage who had sex before marriage and average number of sexual partners, both calculated from individual responses) were used to test whether men assess their risk of paternity uncertainty from the sexual behaviour of men and women in their environment (Vanderende et al., 2016). The DHS records whether others were present during the questions on sexual activity, and individuals to whom this applied were excluded (*n* = 3,921), additionally there were some missing responses for questions concerning sexual activity (*n* = 1,308), giving a total sample size of 20,610.

For hypothesis 2, that men whose reproductive interests conflict with those of their wives will be more likely to commit IPV, the variables used to test reproductive coercion were taken from men and women's stated fertility preferences. Individuals are asked about their desire for more children with the possible responses: more within 2 years; more after 2 years; more but time unspecified; don't know; infertile/sterilised (partner or themselves); or want no more. A comparative variable was calculated from the husbands’ and wives’ responses: both want the same; husband wants more/sooner; wife wants more/sooner; either or both unsure. The variables used to test paternal disinvestment were whether the husband was polygamous, had more living children than his wife, or had had extramarital sex in the preceding 12 months. As marital conflict caused by paternal disinvestment is predicted to vary with how dependent the wife is on her husband for resources, the model also included women's employment status and type of earnings (cash or in-kind). Women who have cash or in-kind earnings are predicted to have a lower risk of IPV. As data exclusions relating to women's sexual activity do not apply, the sample size for this analysis was 25,577.

Data from all 12 countries were pooled and multilevel multivariate logistic regression models were used to examine the association between women's experience of physical IPV and sexual IPV and the independent variables relevant to each hypothesis. Multilevel models deal with hierarchically structured data and partition the sources of behavioural variance at different levels within the model. Ethnic group affinity has been shown to be a strong determinant of individual behaviour (Yoder & Wang, 2013), and a multilevel model structure allows for this clustering at the ethnic group level. Multilevel models recognise the clustering of data at different levels, which addresses some of the perils of analysing aggregate data, and allows assessment of the effect of individual and community effects on the outcome variable as well as estimation of the extent of variation across communities. However, even with large sample sizes of good quality data, this approach does have limitations as individual and ecological variables can be confounded when data are compiled across heterogenous meta-populations (Lawson et al., 2015).

Here three levels were used: individuals (*n* = 25,577 or 20,348), nested within ethnic groups (*n* = 103), nested within countries (*n* = 12). Second- and third-level variance was calculated to understand the variation between ethnic groups and countries regarding the IPV experience (the intercepts in the multilevel logistic regression), and to interpret the importance of the different levels on the outcome. The intra-class correlation, expressed as a percentage, gives a measure of the variance in the logistic outcome attributable to different levels in the model (Goldstein, 2010). Several models were run to test each hypothesis. First, each independent variable was added separately to a model containing just the control variables. Second, an adjusted model was run which included all variables initially shown to be significant in the presence of our control variables alone. Finally, all variables relating to both hypotheses were tested in one model. MLwiN v2.03 was used for multilevel modelling and SPSS v23 was used for other statistical analysis.

## 3. Results

A total 15.9% women reported physical IPV (ranging from 7.7 to 24.8% by country) in the past year and 8.5% reported sexual IPV (ranging from 1.3 to 16.7% by country); overall, 4.4% reported both. Malawi was the only country in which the prevalence of sexual IPV was higher than physical IPV (16.7% compared with 15.7%). Some 55.6% of women who experienced sexual IPV also experienced physical IPV, whereas 29.7% of thise who experienced physical IPV also experienced sexual IPV. Descriptive data for variables used in testing both hypotheses are shown in the Supporting Information (SI) Table S1 and Table S2.

The effect of the control variables on the risk of physical IPV showed that men who drank alcohol, lived in urban areas, agreed with more IPV justifications and who were exposed to IPV in childhood, had higher odds of perpetrating physical IPV. Men's education was not associated with physical IPV perpetration, men's rather than women's age was associated with physical IPV (with younger men at higher risk), and men in the poorest and richest households were least likely to perpetrate physical IPV. Religion was a significant factor, and physical IPV was more likely for couples in which either the husband or wife had more than one marriage. Men who perpetrated sexual IPV were at much higher odds of perpetrating physical IPV.

Fewer control variables were significantly associated with sexual IPV, and where they were significantly associated, the effect size was smaller than with physical IPV (Table 1). Household location, husband's age, wife's age, husband's religion and childhood exposure were not significantly associated with sexual IPV. The husband's use of alcohol, and the number of IPV justifications agreed with, both increased the odds of sexual IPV. Household wealth had a different association with physical IPV, and only women in the richest households had significantly lower odds of experiencing sexual IPV. Men who had perpetrated physical IPV had higher odds of perpetrating sexual IPV.

**Table 1.** Paternity concern: results of fully adjusted multilevel multivariate logistic regression models testing the association between physical and sexual intimate partner violence (IPV) in the past 12 months and variables relating to paternity concern (country, *n* = 12; ethnic groups, *n* = 103; couples, *n* = 20,610)

		Physical IPV				Sexual IPV			
Control variables	Categories	OR	95% CI		*p*-Value	OR	95% CI		*p*-Value
Household wealth (poorest)	Poorer	**1**.**15**	(**1.01–1.32)**		**0**.**039**	0.95	(0.80–1.13)		0.588
	Middle	1.15	(0.10–1.32)		0.055	1.00	(0.84–1.20)		0.971
	Richer	0.92	(0.79–1.08)		0.313	0.94	(0.77–1.15)		0.565
	Richest	0.89	(0.73–1.08)		0.243	**0**.**68**	(**0.52–0.88)**		**0**.**003**
Household residence (urban)	Rural	0.89	(0.78–1.02)		0.065	1.05	(0.89–1.24)		0.579
Husband's education (none)	Primary	1.01	(0.88–1.16)		0.849	0.97	(0.80–1.17)		0.729
	Secondary	1.04	(0.89–1.22)		0.612	0.86	(0.70–1.07)		0.171
	Higher	**0**.**72**	(**0.56–0.93)**		**0**.**010**	0.68	(0.47–0.96)		0.030
Husband's religion (Muslim)	Christian	**0**.**80**	(**0.67–0.95)**		**0**.**010**	0.89	(0.69–1.16)		0.387
	Other/none	0.94	(0.74–1.18)		0.590	0.92	(0.65–1.30)		0.637
Husband's alcohol use (no)	Yes	**2**.**41**	(**2.18–2.67)**		**0**.**000**	**1**.**39**	(**1.22–1.58)**		**0**.**000**
Husband's age (years)		**0**.**98**	(**0.97–0.99)**		**0**.**000**	1.00	(0.99–1.01)		0.789
Wife's age (years)		1.00	(0.99–1.01)		0.521	0.10	(0.98–1.01)		0.709
No. IPV justifications husband agrees with (0–5)		**1**.**08**	(**1.05–1.12)**		**0**.**000**	**1**.**05**	(**1.01–1.10)**		**0**.**024**
Husband ever paid for sex (no)	Yes	1.02	(0.88–1.19)		0.780	**0**.**78**	(**0.64–0.94)**		**0**.**010**
Childhood exposure to IPV (%)		**0**.**10**	(**0.04–0.26)**		**0**.**000**	**0**.**06**	(**0.01–0.24)**		**0**.**016**
Wife: more than one marriage (no)	Yes	0.99	(0.85–1.15)		0.896	1.04	(0.86–1.26)		0.661
Husband: more than one marriage (no)	Yes	**1**.**12**	(**1.00–1.25)**		**0**.**044**	0.94	(0.81–1.08)		0.367
Sexual IPV (no)	Yes	**5**.**67**	(**5.02–6.40)**		**0**.**000**	…	…	…	…
Physical IPV (no)	Yes	…	…	…	…	**5.79**	**(5.11–6.56)**		**0**.**000**
*Independent variables (individual level)*									
Husband jealous (no)	Yes	**1**.**77**	(**1.59–1.96)**		**0**.**000**	**1**.**39**	(**1.21–1.61)**		**0**.**000**
Husband accuses of infidelity (no)	Yes	**2**.**45**	(**2.20–2.72)**		**0**.**000**	**1**.**60**	(**1.40–1.82)**		**0**.**000**
Husband insists on knowing where wife is (no)	Yes	**1**.**29**	(**1.17–1.43)**		**0**.**000**	**2**.**29**	(**2.00–2.62)**		**0**.**000**
Wife: sex before marriage (no)	Yes	1.02	(0.91–1.13)		0.764	0.95	(0.83–1.08)		0.428
Wife: no. sexual partners (one)	Two	**1**.**17**	(**1.03–1.33)**		**0**.**013**	1.01	(0.86–1.19)		0.905
	Three	**1**.**32**	(**1.12–1.57)**		**0**.**001**	**1**.**74**	(**1.41–2.13)**		**0**.**000**
	Four or more	**1**.**37**	(**1.11–1.69)**		**0**.**003**	**1**.**57**	(**1.21–2.03)**		**0**.**001**
*Random part*		*β*	SE	*p*-Value	ICC	*β*	SE	*p*-Value	ICC
Ethnic group		0.14	0.03	0.000	3.8%	0.26	0.06	0.000	6.8%
Country		0.16	0.08	0.045	4.7%	0.26	0.13	0.039	7.2%

Notes: reference categories for categorical variables are shown in brackets.

ICC, Intraclass coefficient, which is a method for measuring the variance explained by each level in the model.

Bold indicates tha the *p*-value is <0.05.

### Hypothesis 1: men exposed to indicators of paternity concern will be more likely to commit IPV

Several of the independent variables are associated with physical and sexual IPV (Table 1). Men who were jealous when their wife talked to another man, accused their wife of unfaithfulness and insisted on knowing where their wife was at all times had significantly higher odds of perpetrating both physical IPV and sexual IPV. Physical IPV was most strongly associated with accusations of unfaithfulness, whereas sexual IPV was more strongly associated with insisting on knowing where his wife was at all times.

Indicators of women's individual sexual activity (premarital sex and number of sexual partners) had a significant association with physical IPV when tested in a model with just the control variables (SI Table S3); however, only lifetime number of sexual partners retained significance in the fully adjusted model (Table 1). Compared with one sexual partner, the risk of physical IPV increased with each extra sexual partner. The woman's number of sexual partners was the only variable significantly associated with sexual IPV in the control and fully adjusted models, and women who had had four or more lifetime sexual partners were at significantly higher risk. None of the indicators of men's individual sexual activity (premarital sex or lifetime number of sexual partners) or the group-level indicators of women and men's sexual activity were associated with the odds of physical or sexual IPV perpetration (SI Table S3), and these variables were not included in the fully adjusted model. Only a small proportion of the country-level and ethnic group-level variation was explained by the fully adjusted models for both physical IPV (3.8 and 4.7%) and sexual IPV (6.8 and 7.2%), which suggests that much of the variation in IPV behaviour was explained by individual rather than group factors. However, the variance remaining is significant, which suggests that further variables not included in the model explain much of the variation.

### Hypothesis 2: men whose reproductive interests conflict with those of their wives will be more likely to commit IPV

The variables testing reproductive coercion relate to the husband and wife's fertility desires. In the model with only the control variables (SI Table S4), men and women's individual desires for more children were not significantly associated with physical IPV or sexual IPV, although women's uncertainty increased the risk of physical IPV. The variable comparing men and women's fertility desires included in the fully adjusted model (Table 2) showed a contrasting effect on the risk of physical IPV or sexual IPV; in line with the prediction, men who wanted more children or sooner than their wives had higher odds of perpetrating physical IPV. However, women who wanted more children or children sooner than their husbands had higher odds of experiencing sexual IPV, a result that is difficult to interpret.

**Table 2.** Reproductive conflict: results of fully adjusted multilevel multivariate logistic regression models testing the association between physical IPV and sexual IPV in the past 12 months and variables relating to reproductive conflict hypothesis (country, *n* = 12; ethnic groups, *n* = 103; couples, *n* = 24,577)

		Physical IPV				Sexual IPV			
Control variables	Categories	OR	95% CI		*p*-Value	OR	95% CI		*p*-Value
Household wealth (poorest)	Poorer	**1**.**14**	**(1.02–1.29)**		**0**.**026**	0.98	(0.84–1.13)		0.747
	Middle	**1**.**16**	**(1.03–1.31)**		**0**.**018**	1.04	(0.89–1.22)		0.638
	Richer	0.95	(0.83–1.09)		0.471	0.90	(0.75–1.07)		0.227
	Richest	0.87	(0.74–1.03)		0.110	**0**.**67**	**(0.54–0.85)**		**0**.**001**
Household residence (urban)	Rural	**0**.**82**	**(0.73–0.91)**		**0**.**001**	0.96	(0.83–1.11)		0.600
Husband's education (none)	Primary	1.07	(0.95–1.21)		0.277	1.11	(0.94–1.31)		0.234
	Secondary	1.09	(0.95–1.25)		0.208	0.96	(0.80–1.16)		0.686
	Higher	**0**.**75**	**(0.61–0.94)**		**0**.**010**	0.79	(0.58–1.09)		0.145
Husband's religion (Muslim)	Christian	**0**.**82**	**(0.70–0.95)**		**0**.**009**	0.94	(0.74–1.19)		0.587
	Other/none	0.90	(0.73–1.10)		0.296	0.96	(0.74–1.31)		0.797
Husband drinks alcohol (no)	Yes	**2**.**66**	**(2.44–2.92)**		**0**.**000**	**1**.**53**	**(1.36–1.72)**		**0**.**000**
No. IPV reasons husband agrees with (0–5)		**1**.**07**	**(1.04–1.11)**		**0**.**000**	**1**.**07**	**(1.03–1.11)**		**0**.**001**
Husband's age (years)		**0**.**98**	**(0.97–0.98)**		**0**.**000**	1.00	(0.99–1.01)		0.579
Wife's age (years)		1.00	(1.00–1.02)		0.242	1.00	(0.99–1.01)	0.637	
Childhood exposure to IPV (%)		**1**.**03**	**(1.02–1.04)**		**0**.**000**	1.01	(1.00–1.03)		0.067
Sexual IPV (No)	Yes	**7**.**80**	**(7.02–8.67)**		**0**.**000**	…	…	…	…
Physical IPV (No)	Yes	…	…	…	…	**8.00**	**(7.17–8.93)**		**0.000**
*Independent variables: reproductive coercion*									
Fertility desire comparison (Both want no more)	Disagree: husband wants more/sooner	**1**.**12**	**(1.02**	**–1.24)**	**0**.**025**	1.02	(0.89	–1.16)	0.792
	Disagree: wife wants more/sooner	1.08	(0.96	–1.20)	0.195	**1.21**	**(1**.**05**	**–1.40)**	**0**.**008**
	Either unsure	0.97	(0.83	–1.12)	0.628	0.87	(0.71	–1.06)	0.156
*Independent variables: paternal disinvestment*									
Living children comparison (both have the same)	Wife has more	1.09	(0.94	–1.25)	0.249	1.15	(0.96	–1.38)	0.136
	Husband has more	**1**.**13**	**(1.02**	**–1.24)**	**0**.**015**	**1.15**	**(1**.**02**	**–1.29)**	**0**.**020**
Polygamous (no)	Yes	**1**.**24**	**(1.09**	**–1.42)**	**0**.**001**	0.99	(0.83	–1.19)	0.946
Husband had extramarital sex last 12 months (no)	Yes	**1**.**32**	**(1.16**	**–1.49)**	**0**.**000**	1.07	(0.91	–1.27)	0.410
Wife's economic independence (not working)	Works but not paid	0.91	(0.81	–1.02)	0.092	**1.16**	**(1**.**35**	**–1.80)**	**0**.**000**
	Paid in cash	1.03	(0.93	–1.14)	0.567	**1.66**	**(1**.**02**	**–1.36)**	**0**.**028**
	Paid in cash/kind	1.08	(0.94	–1.25)	0.262	**1.17**	**(1**.**37**	**–2.01)**	**0**.**000**
*Random part*		*β*	SE	*p*-Value	ICC	*β*	SE	*p*-Value	ICC
Ethnic group		0.12	0.03	0.000	3.4%	0.24	0.05	0.000	6.1%
Country		0.15	0.07	0.037	4.2%	0.43	0.20	0.033	11.6%

Notes: Reference categories for categorical variables are shown in brackets.

Bold indicates that the *p*-value is <0.05.

The paternal disinvestment variables were significantly associated with physical IPV. The fully adjusted model (Table 2) shows that the risk of physical IPV was higher in marriages where the husband had more living children than their wife, was polygamous and had had extramarital sex within the past year. Interactions between marital type, extramarital sex incidence and difference in living children were run to assess whether men who were polygamous and had had extramarital sex, may also have had more living children than their wives. The interactions were not significant, which suggests that the same individuals were not causing the statistical effect. Women's economic independence does not have a protective effect against physical IPV. In contrast, the odds of sexual IPV were not higher for polygamous men or men who had had extramarital sex, but men with more living children than their wives were shown to be more likely to perpetrate sexual IPV in the fully adjusted model. Women in employment were at higher risk of sexual IPV compared with women who were not working. This is irrespective of whether they were not paid, paid in cash, or paid in a mixture of cash and kind.

The fully adjusted model (Table 2) showed that the variation explained at the country and ethnic group level is higher for the sexual IPV model than for the physical IPV models (a total of 17.7% compared with 7.6%), which suggests that more of the variation in physical IPV is explained by individual-level factors. A combined model in which all variables from the fully adjusted models testing both paternity concern and reproductive conflict were included in one model (Table 3) showed most variables retaining significance in both the physical IPV and sexual IPV models, the only exception being the number of living children.

**Table 3.** Paternity concern and reproductive conflict. Results of multilevel multivariate logistic regression analysis testing the association between physical IPV and sexual IPV in the past 12 months and variables relating to paternity concern and reproductive conflict hypotheses (country, *n* = 12; ethnic groups, *n* = 103; couples, *n* = 20,610)

		Physical IPV				Sexual IPV			
Control variables	Categories	OR	95% CI		*p*-Value	OR	95% CI		*p*-Value
Household wealth (poorest)	Poorer	1.14	(0.99–1.30)		0.066	0.97	(0.81–1.15)		0.712
	Middle	1.12	(0.97–1.29)		0.131	1.027	(0.85–1.23)		0.797
	Richer	0.90	(0.76–1.05)		0.187	0.99	(0.81–1.21)		0.912
	Richest	0.86	(0.70–1.05)		0.126	**0**.**70**	(**0.54–0.91)**		**0**.**008**
Household residence (urban)	Rural	0.89	(0.78–1.01)		0.065	1.02	(0.86–1.20)		0.859
Husband's education (none)	Primary	1.02	(0.88–1.17)		0.820	0.95	(0.79–1.15)		0.599
	Secondary	1.04	(0.89–1.22)		0.599	0.85	(0.69–1.06)		0.142
	Higher	**0**.**72**	(**0.56–0.93)**		**0**.**010**	**0**.**68**	(**0.47–0.97)**		**0**.**034**
Husband's religion (Muslim)	Christian	**0**.**80**	(**0.67–0.95)**		**0**.**010**	0.84	(0.65–1.09)		0.194
	Other/none	0.93	(0.73–1.17)		0.515	0.87	(0.61–1.24)		0.435
Husband drinks alcohol (no)	Yes	**2**.**40**	(**2.17–2.66)**		**0**.**000**	**1**.**39**	(**1.22–1.59)**		**0**.**000**
No. IPV justifications husband agrees with (0–5)		**1**.**08**	(**1.05–1.12)**		**0**.**000**	**1**.**05**	(**1.01–1.10)**		**0**.**022**
Husband ever paid for sex (no)	Yes	1.00	(0.86–1.16)		0.968	**0**.**75**	(**0.62–0.92)**		**0**.**005**
Husband's age (years)		**0**.**98**	(**0.97–0.99)**		**0**.**000**	1.00	(0.99–1.02)		0.698
Wife's age (years)		1.00	(0.99–1.01)		0.422	1.00	(0.98–1.01)		0.535
Childhood exposure (% in ethnic group)		**1**.**03**	(**1.02–1.04)**		**0**.**000**	**1**.**02**	(**1.00–1.03)**		**0**.**046**
Wife: one or more marriages (one)	More than one	1.01	(0.86–1.17)		0.934	1.07	(0.88–1.30)		0.474
Husband: one or more marriages (one)	More than one	1.05	(0.92–1.21)		0.456	0.96	(0.81–1.14)		0.643
Sexual IPV (no)	Yes	**5**.**70**	(**5.03–6.44)**		**0**.**000**	…	…	…	…
Physical IPV (no)	Yes	…	…	…	…	**5.91**	**(5.20–6.71)**		**0**.**000**
*Independent variables: paternity concern*									
Husband jealous (no)	Yes	**1**.**74**	(**1.57–1.93)**		**0**.**000**	**1**.**40**	(**1.21–1.63)**		**0**.**000**
Husband accuses of infidelity (no)	Yes	**2**.**46**	(**2.22–2.74)**		**0**.**000**	**1**.**60**	(**1.40–1.82)**		**0**.**000**
Husband insists knowing where wife is (no)	Yes	**1**.**29**	(**1.17–1.43)**		**0**.**000**	**2**.**25**	(**1.96–2.57)**		**0**.**000**
Wife: no. sexual partners in lifetime (One)	Two	**1**.**19**	(**1.06–1.34)**		**0**.**003**	0.98	(0.84–1.15)		0.805
	Three	**1**.**36**	(**1.16–1.59)**		**0**.**000**	**1**.**70**	(**1.40–2.07)**		**0**.**000**
	Four or more	**1**.**36**	(**1.05–1.76)**		**0**.**019**	**1**.**86**	(**1.36–2.53)**		**0**.**000**
*Independent variables: reproductive coercion*									
Fertility desire comparison (both want no more)	Disagree: husband wants more/sooner	**1**.**13**	(**1.01–1.27)**		**0**.**035**	1.08	(0.93–1.26)		0.312
	Disagree: wife wants more/sooner	1.06	(0.94–1.21)		0.356	**1**.**23**	(**1.04–1.45)**		**0**.**014**
	Either unsure	0.92	(0.78–1.10)		0.367	0.87	(0.70–1.09)		0.234
*Independent variables: paternal disinvestment*									
Living children comparison (both have the same)	Wife has more	0.95	(0.80–1.14)		0.574	0.94	(0.75–1.18)		0.613
	Husband has more	1.02	(0.90–1.15)		0.748	1.03	(0.88–1.21)		0.703
Polygamous (no)	Yes	**1**.**20**	(**1.01–1.42)**		**0**.**037**	0.90	(0.72–1.12)		0.345
Husband had extramarital sex last 12 months (no)	Yes	**1**.**22**	(**1.05–1.41)**		**0**.**008**	1.12	(0.92–1.35)		0.262
Wife's economic independence (not working)	Works but not paid	0.90	(0.79–1.03)		0.119	**1**.**55**	(**1.32–1.83)**		**0**.**000**
	Paid in cash	1.03	(0.92–1.16)		0.608	**1**.**18**	(**1.01–1.38)**		**0**.**041**
	Paid in cash/kind	1.09	(0.93–1.28)		0.308	**1**.**67**	(**1.35–2.07)**		**0**.**000**
*Random part*		*β*	SE	*p*-Value	ICC	*β*	SE	*p*-Value	ICC
Country		0.15	0.07	0.000	4%	0.32	0.16	0.000	9%
ethnic group		0.13	0.03	0.006	4%	0.26	0.06	0.000	7%

Notes: reference categories for categorical variables are shown in brackets.

Bold indicates that the *p*-value is <0.05.

## 4. Discussion

This study tested two evolutionary theories for male-to-female IPV perpetration using couple data from 12 countries in sub-Saharan Africa; firstly, whether men exposed to indicators of paternity concern are more likely to commit IPV; and secondly, whether men whose reproductive interests conflict with those of their wives will be more likely to commit IPV. The results provide evidence that perpetrating physical IPV is associated with indicators of paternity concern and indicators of reproductive conflict (specifically paternal disinvestment) whereas sexual IPV perpetration is only associated with indicators of paternity concern. The large and detailed datasets used mean that numerous factors known to be confounded with IPV perpetration, such as those associated with living in a marginal environment and ethnic variation, were controlled for.

Paternity concern has been put forward as an explanation for a wide range of male behaviours, such as mate-guarding, coercive control and IPV; however, few studies have demonstrated this empirically (Hartung, 1985; Buss, 1996; Goetz et al., 2008). Our results indicate that paternity concern is a motivating factor for IPV in this sample (Tables 1 and 3), finding that men are more likely to perpetrate IPV when the risk of their wife engaging in extra-pair sex may be perceived to be higher. Firstly, men who were reported to exhibit jealousy, make accusations of infidelity, and insist on knowing where their wife is at all times, had significantly higher odds of perpetrating both physical and sexual IPV. The association with physical and sexual IPV is similar, which supports the prediction that all types of IPV, not just sexual IPV, may be forms of sexual coercion related to men's concern about their wives’ infidelity (Goetz & Shackelford, 2009). Secondly, only indicators of the wife's sexual activity (rather than that of others in the group) are associated with an increased risk of physical and sexual IPV. This suggests that in this context men are primarily attuned to their wives’ sexual behaviour and their own personal risk of non-paternity, rather than a more general threat posed by the behaviour of others in the group.

The statistical model examining reproductive conflict tested two motivations for men perpetrating IPV: achieving higher fertility either within their marriage (reproductive coercion) or outside their marriage (paternal disinvestment) (Table 2). The evidence that men use IPV to achieve higher fitness within their marriage is weak; however, stronger support for the paternal disinvestment hypothesis is found, which predicts that conflict arises when men seek to increase their fitness outside their marriage (Stieglitz et al., 2011). The odds of physical IPV are significantly higher for men who have more living children than their wives, are polygamous and have reported having extramarital sex in the past year. These are all situations in which men may be diverting resources away from their wives’ households, potentially enhancing their own fitness to the detriment of their wives’. Paternal disinvestment theory predicts that marital conflict escalates into physical IPV, and accordingly, fewer associations are found between paternal disinvestment proxies and sexual IPV (Stieglitz et al., 2011). However contrary to this prediction, women in employment – who are anticipated to be less financially reliant on their husbands with less cause for ‘resource’ jealousy – do not have lower odds of experiencing physical IPV, and are actually at higher risk of sexual IPV. Rather than providing economic independence, women's earnings may be a trigger for conflict within a marriage in line with the relative resource social theory for IPV (Allen & Straus, 1975; Vyas & Watts, 2009). A proper test of relative resource theory requires data on the relative income and education discrepancies between men and women, not simply women's status (Kilgallen et al., 2021). An alternative interpretation could be that employment results in women's absence from the home, which triggers IPV motivated by paternity concern.

The higher odds of physical IPV in polygamous marriages is in line with previous studies (e.g. in Kenya Makayoto et al., 2013; Kimuna & Djamba, 2008). While the practice of polygynous marriage varies across cultures, these results give some further support to paternal disinvestment theory rather than reproductive coercion theory as a motivating factor for IPV in this context (Stieglitz et al., 2011); women in polygamous marriages may be faced with paternal disinvestment regularly, as their husbands invest resources on co-wives and their co-wives’ offspring.

These different evolutionary motivations for IPV are not anticipated to be mutually exclusive, and the full model (Table 3) shows that when all proxy indicators for both paternity certainty and reproductive conflict are included in the same model, they retain the same association with IPV perpetration. The relative importance of these different motivations are likely to vary depending on a range of ecological factors, such as the availability of alternative partners and cultural norms concerning paternal investment (Borgerhoff Mulder & Rauch, 2009). To distinguish between these evolutionary motivations, genetic testing and data on men's reproductive success would be required; it would be anticipated that the fitness benefit gained by IPV triggered by paternity concern would be a reduction in men's non-paternity rate (Buss, 1996), whereas the fitness gained by paternal disinvestment would be an increase in men's own fertility (Stieglitz et al., 2018).

The results also provide an insight into the relationship between physical and sexual IPV. There is evidently a strong overlap, with the experience of physical IPV being more strongly associated with experience of sexual IPV than in the opposition direction. However, the two behaviours do not always co-occur and during the same 12 month period 44.3% of women who experienced sexual IPV did not experience physical IPV as well (955 out of 2153 women). In line with previous studies we find that ewer variables are significantly associated with sexual IPV than physical IPV (Townsend et al., 2011; Fulu et al., 2013) The results also reveal two novel findings. Firstly, sexual IPV is associated with fewer proxy indicators for evolutionary motivations than physical IPV. This may reflect the lower incidence of sexual IPV, which reduces the power of the analysis, but may also indicate that sexual IPV serves no fitness benefit. Intimate parner violence of all types carry numerous evolutionary costs for the perpetrator, and it is plausible that sexual IPV as measured by the DHS carries higher costs than physical IPV (which can refer to a range of behaviours of varying severity), and men may achieve higher fitness in their intimate partnerships by using less costly strategies. Secondly, sexual IPV is not significantly associated with indicators of paternal disinvestment, and is more strongly associated with indicators of paternity concern (the number of the wife's sexual partners) than physical IPV. These results suggest different motivations for physical and sexual IPV perpetration, highlighting the need for different intervention strategies. Current intervention strategies do not typically distinguish between IPV types (Devries et al., 2013b).

The ultimate-level explanations tested here are not alternatives to proximate explanations, and the outcomes are often aligned (Tinbergen, 1963; Scott-Phillips et al., 2011). For example, a wife's extra-pair sex could be a proximate trigger for IPV, and also provide an ultimate-level motivation if this jeopardises her husband's evolutionary fitness. Proving that IPV perpetration has an ultimate motivation and that it enhances men's evolutionary fitness requires data demonstrating that men gain a fitness benefit from their behaviour. To date this has only been tested directly in one evolutionary study which found that physical IPV perpetration predicts higher marital fertility (sexual IPV was not tested; Stieglitz et al., 2018). Further, alternative evolutionary motivations not tested here may also contribute to IPV behaviours. Not all men perpetrate IPV even in societies in which IPV is commonplace, which suggests that alternative fitness-enhancing strategies exist. Shackelford et al. (2002) have looked at positive male attentiveness to their partners as an alternative to violence.

The study does have some important limitations. Firstly, as cross-sectional data is used, the precise sequence of events between the IPV incidents and some of the variables is not known. The variables were matched as closely as possible to the timeframe of the IPV incident, and the large sample size compensates somewhat for the lack of detailed time-sequence events; however, there is still ambiguity for some variables. Further, the frequency of each type of violence is not known, and the resulting measurement is binary (did/did not experience IPV) rather than quantitative (WHO/LSHTM, 2010). There are differences of opinion whether a single instance of violence constitutes IPV, and regarding the comparability of sexual and physical IPV measures captured here (Heise, 2012). Further work in capturing IPV intensity or measuring the nuances of different IPV experiences is required. Secondly, sexual activity and IPV data are self-reported and highly sensitive, and therefore subject to reporting bias. The DHS tries to minimise reporting bias, for example using interviewers specifically trained in asking about IPV, and to reduce recall errors IPV experience was restricted to the prior 12 months in this study; however, IPV instances may be underreported and under-represented (Gibson et al, 2022). Thirdly, a large sample size increases statistical power, enabling us to detect even small associations between variables. However, future research could pay greater attention to issues of effect size. Our model selection approach is also simple, and could be better informed by the potential causal relationships between independent and control variables to better estimate causality. Fourthly, the multilevel approach used here, which combines data across regional and ethnic contexts, does not provide a thorough understanding of how context matters, as only the main effects of each variable are considered. Future work could attempt to incorporate contextual factors, potentially from ethnologically grounded anthropological studies. Finally, here we have identified important relationships which are consistent with our hypotheses; however, there may be further ecological confounding factors which covary with IPV and the experiment variables which we have not been able to fully control for. Future work could explore the underlying associations and relationships between these variables to reveal potential confounds.

## 5. Conclusion

In this study multilevel logistic regression models using couples’ data from 12 sub-Saharan African countries were used to test evolutionary explanations for male-to-female IPV perpetration, analysing physical and sexual IPV outcomes separately. Evolutionary theorists have argued that IPV reflects conflict between the sexes, arising from men and women's differing fitness goals (Borgerhoff Mulder & Rauch, 2009; Parker, 1979). Two hypotheses based on this theory were tested: (a) men perpetrate IPV in response to their wives’ actual or perceived risk of extra-pair sex; and (b) IPV occurs owing to reproductive conflict, leading men to pursue higher fertility than women, either within marriage (reproductive coercion) or with alternative partners outside marriage (paternal disinvestment)

The results show support for both hypotheses, differing by IPV type. Indicators of paternity concern increase the odds of both physical and sexual IPV perpetration. An evolutionary interpretation is that men are attuned to their partner's sexual behaviour and their own specific risk of non-paternity, and perpetrate IPV where the perceived risk is higher (Goetz et al., 2008). Indicators of reproductive conflict (resulting from men having a higher optimum fitness than women), specifically indicators of paternal disinvestment, show a stronger association with physical IPV but not sexual IPV perpetration. Men who are polygamous, have more living children than their wife and engage in extramarital sex are more likely to perpetrate physical IPV. An evolutionary interpretation is that marital conflict over paternal investment being diverted from the wife's family unit is evidence of men and women's differing evolutionary goals. To compensate for the considerable fitness costs associated with men perpetrating IPV, it is predicted that men will be more likely to ‘disinvest’ in certain contexts, e.g. where there is a greater availability of alternative mates or greater social acceptance of changing partners (Borgerhoff Mulder & Rauch, 2009; Stieglitz et al., 2018). Finally, it is worth noting that our data represent a range of differing cultural ecologies which may explain the observed variation in IPV behaviour. Previous studies have demonstrated that cross-cultural variation in male behaviour may be predicted by different relationship norms in their local social ecology (Scelza et al., 2020).

These results demonstrate that an evolutionary approach can enhance our understanding of male-to-female IPV, complementing the knowledge gained from non-evolutionary studies (Gibson & Lawson, 2014). The variables used here to test both evolutionary theories have been shown in previous studies to be associated with IPV. Here these variables are placed in an evolutionary framework which gives context and explanation for why these factors might increase the risk of IPV. An evolutionary approach goes beyond the theory that men perpetrate IPV to control their partners, and seeks to explain why they might be driven to do so.

## Research transparency and reproducibility

The data used in this study is publicly available via https://dhsprogram.com/

## Supplementary material

For supplementary material accompanying this paper visit https://doi.org/10.1017/ehs.2022.58. click here to view supplementary material
