# Supplementary material for: Testing evolutionary conflict theories for sexual and physical intimate partner violence in Sub-Saharan Africa
Source: Evol Hum Sci. 2022 Dec 19;5:e6. doi: 10.1017/ehs.2022.58 (PMC10426027; doi:10.1017/ehs.2022.58)
Supplement: Supplementary file 1 [file S2513843X22000585sup001.docx]

**TESTING EVOLUTIONARY CONFLICT THEORIES FOR SEXUAL AND PHYSICAL INTIMATE PARTNER VIOLENCE IN SUB-SAHARAN AFRICA**

**SUPPEMENTARY INFORMATION**

**Table 1. Frequency of IPPV and IPSV for variables used to test paternity concern hypotheses (n 20,610)**

|  |  | **IPPV** | **% of IPPV** | **IPSV** | **% of IPSV** | **TOTAL** | **% of TOTAL** |
| --- | --- | --- | --- | --- | --- | --- | --- |
|  | **Total** | **3252** | **15.8%** | **1757** | **8.5%** | **20610** | **100.0%** |
|  |  |  |  |  |  |  |  |
| Country | Burkina Faso | 215 | 9.5% | 29 | 1.3% | 2268 | 11.0% |
|  | Chad | 137 | 13.5% | 73 | 7.2% | 1012 | 4.9% |
|  | Ethiopia | 263 | 14.0% | 114 | 6.1% | 1877 | 9.1% |
|  | Gambia | 30 | 8.2% | 5 | 1.4% | 367 | 1.8% |
|  | Ghana | 131 | 18.0% | 34 | 4.7% | 726 | 3.5% |
|  | Ivory Coast | 113 | 22.7% | 20 | 4.0% | 497 | 2.4% |
|  | Kenya | 378 | 24.7% | 158 | 10.3% | 1531 | 7.4% |
|  | Malawi | 409 | 15.3% | 441 | 16.5% | 2668 | 12.9% |
|  | Mali | 218 | 21.1% | 108 | 10.5% | 1031 | 5.0% |
|  | Nigeria | 261 | 7.8% | 98 | 2.9% | 3342 | 16.2% |
|  | Togo | 172 | 14.0% | 64 | 5.2% | 1228 | 6.0% |
|  | Zambia | 925 | 22.8% | 613 | 15.1% | 4063 | 19.7% |
| **CATEGORICAL CONTROL VARIABLES** | |  |  |  |  |  |  |
| Household wealth | Poorest | 690 | 15.7% | 398 | 9.0% | 4406 | 21.4% |
|  | Poorer | 770 | 17.7% | 427 | 9.8% | 4354 | 21.1% |
|  | Middle | 721 | 18.0% | 408 | 10.2% | 3999 | 19.4% |
|  | Richer | 587 | 14.8% | 324 | 8.2% | 3963 | 19.2% |
|  | Richest | 484 | 12.4% | 200 | 5.1% | 3888 | 18.9% |
| Household residence | Urban | 989 | 15.8% | 451 | 7.2% | 6272 | 30.4% |
|  | Rural | 2263 | 15.8% | 1306 | 9.1% | 14338 | 69.6% |
| Education (husband) | No education | 757 | 12.1% | 350 | 5.6% | 6252 | 30.3% |
|  | Primary | 1362 | 19.0% | 843 | 11.7% | 7187 | 34.9% |
|  | Secondary | 989 | 17.5% | 502 | 8.9% | 5651 | 27.4% |
|  | Higher | 144 | 9.5% | 62 | 4.1% | 1520 | 7.4% |
| Religion (husband) | Muslim | 747 | 10.4% | 337 | 4.7% | 7194 | 34.9% |
|  | Christian | 2254 | 18.5% | 1323 | 10.8% | 12207 | 59.2% |
|  | Other/none | 251 | 20.8% | 97 | 8.0% | 1209 | 5.9% |
| Alcohol use (husband) | No | 1570 | 11.0% | 914 | 6.4% | 14336 | 69.6% |
|  | Yes | 1682 | 26.8% | 843 | 13.4% | 6274 | 30.4% |
| Transactional sex (husband) | No | 2872 | 15.4% | 1560 | 8.4% | 18612 | 90.3% |
|  | Yes | 351 | 19.8% | 193 | 10.9% | 1776 | 8.6% |
| Number of unions (wife) | Once | 2741 | 15.1% | 1444 | 8.0% | 18098 | 87.8% |
|  | More than once | 511 | 20.3% | 313 | 12.5% | 2512 | 12.2% |
| Number of unions (husband) | Once | 2198 | 15.2% | 1169 | 8.1% | 14490 | 70.3% |
|  | More than once | 1054 | 17.2% | 588 | 9.6% | 6120 | 29.7% |
| Experienced IPPV last 12m | No | .. | .. | 791 | 4.6% | 17358 | 84.2% |
|  | Yes | .. | .. | 966 | 29.7% | 3252 | 15.8% |
| Experienced IPSV last 12m | No | 2286 | 12.1% | .. | .. | 18853 | 91.5% |
|  | Yes | 966 | 55.0% | .. | .. | 1757 | 8.5% |
| **CATEGORICAL INDEPENDENT VARIABLES** | |  |  |  |  |  |  |
| Jealous wife if talks to other men | No | 868 | 8.7% | 425 | 4.3% | 9947 | 48.3% |
|  | Yes | 2359 | 22.5% | 1317 | 12.6% | 10492 | 50.9% |
| Accuses wife of unfaithfulness | No | 1858 | 11.0% | 943 | 5.6% | 16890 | 82.0% |
|  | Yes | 1379 | 38.0% | 805 | 22.2% | 3626 | 17.6% |
| Insists knowing where she is at all times | No | 1297 | 10.6% | 488 | 4.0% | 12199 | 59.2% |
|  | Yes | 1946 | 23.3% | 1265 | 15.1% | 8351 | 40.5% |
| Wife: sex before marriage | No | 1903 | 14.1% | 1017 | 7.5% | 13535 | 65.7% |
|  | Yes | 1349 | 19.1% | 740 | 10.5% | 7075 | 34.3% |
| Wife: lifetime number of sexual partners | 1 | 1688 | 13.1% | 879 | 6.8% | 12892 | 62.6% |
|  | 2 | 894 | 18.0% | 457 | 9.2% | 4969 | 24.1% |
|  | 3 | 427 | 23.3% | 280 | 15.3% | 1829 | 8.9% |
|  | 4+ | 243 | 26.4% | 141 | 15.3% | 920 | 4.5% |
| Husband: sex before marriage | No | 908 | 12.8% | 478 | 6.8% | 7072 | 34.3% |
|  | Yes | 2344 | 17.3% | 1279 | 9.4% | 13538 | 65.7% |
| Husband: lifetime number of sexual partners | 1 | 455 | 10.7% | 239 | 5.6% | 4250 | 20.6% |
|  | 2 | 612 | 14.6% | 351 | 8.3% | 4206 | 20.4% |
|  | 3 | 556 | 16.3% | 303 | 8.9% | 3413 | 16.6% |
|  | 4 + | 1629 | 18.4% | 864 | 9.8% | 8741 | 21.3% |
|  |  |  |  |  |  |  |  |
| **Table 1 cont.** |  | **IPPV** |  | **IPSV** |  | **TOTAL** |  |
|  |  | **Mean** | **(sd)** | **Mean** | **(sd)** | **Mean** | **(sd)** |
|  |  |  |  |  |  |  |  |
| **SCALE CONTROL VARIABLES** |  |  |  |  |  |  |  |
| Wife's current age |  | 29.9 | 7.3 | 30.0 | 7.2 | 30.2 | 7.6 |
| Husband's current age |  | 36.0 | 8.2 | 36.0 | 8.2 | 37.0 | 8.5 |
| Number of IPV justifications agreed with by husband | |  |  |  |  |  |  |
| % father beat mother in husband ethnic group | | 29% | 14% | 31% | 13% | 23% | 15% |
|  |  |  |  |  |  |  |  |
| **SCALE INDEPENDENT VARIABLES** | |  |  |  |  |  |  |
| Wife: average number sexual partners in ethnic group | | 1.9 | 0.5 | 1.9 | 0.4 | 1.7 | 0.5 |
| Wife: prevalence of sex before marriage in ethnic group | | 40% | 18% | 42% | 17% | 35% | 20% |
| Husband: average number sexual partners in ethnic group | | 5.7 | 2.4 | 5.5 | 2.2 | 5.1 | 2.4 |
| Husband: prevalence of sex before marriage in ethnic group | | 72% | 18% | 72% | 18% | 66% | 23% |
|  |  |  |  |  |  |  |  |

**Table 2. Frequency of IPPV and IPSV for variables used to test reproductive conflict hypotheses (n25,577)**

|  |  | **IPPV** | **% of IPPV** | **IPSV** | **% of IPSV** | **TOTAL** | **% of TOTAL** |
| --- | --- | --- | --- | --- | --- | --- | --- |
|  | **Total** | **3252** | **15.8%** | **1757** | **8.5%** | **20610** | **100.0%** |
|  |  |  |  |  |  |  |  |
| Country | Burkina Faso | 215 | 9.5% | 29 | 1.3% | 2268 | 11.0% |
|  | Chad | 137 | 13.5% | 73 | 7.2% | 1012 | 4.9% |
|  | Ethiopia | 263 | 14.0% | 114 | 6.1% | 1877 | 9.1% |
|  | Gambia | 30 | 8.2% | 5 | 1.4% | 367 | 1.8% |
|  | Ghana | 131 | 18.0% | 34 | 4.7% | 726 | 3.5% |
|  | Ivory Coast | 113 | 22.7% | 20 | 4.0% | 497 | 2.4% |
|  | Kenya | 378 | 24.7% | 158 | 10.3% | 1531 | 7.4% |
|  | Malawi | 409 | 15.3% | 441 | 16.5% | 2668 | 12.9% |
|  | Mali | 218 | 21.1% | 108 | 10.5% | 1031 | 5.0% |
|  | Nigeria | 261 | 7.8% | 98 | 2.9% | 3342 | 16.2% |
|  | Togo | 172 | 14.0% | 64 | 5.2% | 1228 | 6.0% |
|  | Zambia | 925 | 22.8% | 613 | 15.1% | 4063 | 19.7% |
| **CATEGORICAL CONTROL VARIABLES** | |  |  |  |  |  |  |
| Household wealth | Poorest | 690 | 15.7% | 398 | 9.0% | 4406 | 21.4% |
|  | Poorer | 770 | 17.7% | 427 | 9.8% | 4354 | 21.1% |
|  | Middle | 721 | 18.0% | 408 | 10.2% | 3999 | 19.4% |
|  | Richer | 587 | 14.8% | 324 | 8.2% | 3963 | 19.2% |
|  | Richest | 484 | 12.4% | 200 | 5.1% | 3888 | 18.9% |
| Household residence | Urban | 989 | 15.8% | 451 | 7.2% | 6272 | 30.4% |
|  | Rural | 2263 | 15.8% | 1306 | 9.1% | 14338 | 69.6% |
| Education (husband) | No education | 757 | 12.1% | 350 | 5.6% | 6252 | 30.3% |
|  | Primary | 1362 | 19.0% | 843 | 11.7% | 7187 | 34.9% |
|  | Secondary | 989 | 17.5% | 502 | 8.9% | 5651 | 27.4% |
|  | Higher | 144 | 9.5% | 62 | 4.1% | 1520 | 7.4% |
| Religion (husband) | Muslim | 747 | 10.4% | 337 | 4.7% | 7194 | 34.9% |
|  | Christian | 2254 | 18.5% | 1323 | 10.8% | 12207 | 59.2% |
|  | Other/none | 251 | 20.8% | 97 | 8.0% | 1209 | 5.9% |
| Alcohol use (husband) | No | 1570 | 11.0% | 914 | 6.4% | 14336 | 69.6% |
|  | Yes | 1682 | 26.8% | 843 | 13.4% | 6274 | 30.4% |
| Transactional sex (husband) | No | 2872 | 15.4% | 1560 | 8.4% | 18612 | 90.3% |
|  | Yes | 351 | 19.8% | 193 | 10.9% | 1776 | 8.6% |
| Number of unions (wife) | Once | 2741 | 15.1% | 1444 | 8.0% | 18098 | 87.8% |
|  | More than once | 511 | 20.3% | 313 | 12.5% | 2512 | 12.2% |
| Number of unions (husband) | Once | 2198 | 15.2% | 1169 | 8.1% | 14490 | 70.3% |
|  | More than once | 1054 | 17.2% | 588 | 9.6% | 6120 | 29.7% |
| Experienced IPPV last 12m | No | .. | .. | 791 | 4.6% | 17358 | 84.2% |
|  | Yes | .. | .. | 966 | 29.7% | 3252 | 15.8% |
| Experienced IPSV last 12m | No | 2286 | 12.1% | .. | .. | 18853 | 91.5% |
|  | Yes | 966 | 55.0% | .. | .. | 1757 | 8.5% |
| **CATEGORICAL INDEPENDENT VARIABLES** | |  |  |  |  |  |  |
| Jealous wife if talks to other men | No | 868 | 8.7% | 425 | 4.3% | 9947 | 48.3% |
|  | Yes | 2359 | 22.5% | 1317 | 12.6% | 10492 | 50.9% |
| Accuses wife of unfaithfulness | No | 1858 | 11.0% | 943 | 5.6% | 16890 | 82.0% |
|  | Yes | 1379 | 38.0% | 805 | 22.2% | 3626 | 17.6% |
| Insists knowing where she is at all times | No | 1297 | 10.6% | 488 | 4.0% | 12199 | 59.2% |
|  | Yes | 1946 | 23.3% | 1265 | 15.1% | 8351 | 40.5% |
| Wife: sex before marriage | No | 1903 | 14.1% | 1017 | 7.5% | 13535 | 65.7% |
|  | Yes | 1349 | 19.1% | 740 | 10.5% | 7075 | 34.3% |
| Wife: lifetime number of sexual partners | 1 | 1688 | 13.1% | 879 | 6.8% | 12892 | 62.6% |
|  | 2 | 894 | 18.0% | 457 | 9.2% | 4969 | 24.1% |
|  | 3 | 427 | 23.3% | 280 | 15.3% | 1829 | 8.9% |
|  | 4+ | 243 | 26.4% | 141 | 15.3% | 920 | 4.5% |
| Husband: sex before marriage | No | 908 | 12.8% | 478 | 6.8% | 7072 | 34.3% |
|  | Yes | 2344 | 17.3% | 1279 | 9.4% | 13538 | 65.7% |
| Husband: lifetime number of sexual partners | 1 | 455 | 10.7% | 239 | 5.6% | 4250 | 20.6% |
|  | 2 | 612 | 14.6% | 351 | 8.3% | 4206 | 20.4% |
|  | 3 | 556 | 16.3% | 303 | 8.9% | 3413 | 16.6% |
|  | 4 + | 1629 | 18.4% | 864 | 9.8% | 8741 | 21.3% |

| **Table 2 cont.** |  | **IPPV** |  | **IPSV** |  | **TOTAL** |  |
| --- | --- | --- | --- | --- | --- | --- | --- |
|  |  | **Mean** | **(sd)** | **Mean** | **(sd)** | **Mean** | **(sd)** |
|  |  |  |  |  |  |  |  |
| **SCALE CONTROL VARIABLES** |  |  |  |  |  |  |  |
| Wife's current age |  | 29.9 | 7.3 | 30.0 | 7.2 | 30.2 | 7.6 |
| Husband's current age |  | 36.0 | 8.2 | 36.0 | 8.2 | 37.0 | 8.5 |
| Number of IPV justifications agreed with by husband | |  |  |  |  |  |  |
| % father beat mother in husband ethnic group | | 29% | 14% | 31% | 13% | 23% | 15% |
|  |  |  |  |  |  |  |  |
| **SCALE INDEPENDENT VARIABLES** | |  |  |  |  |  |  |
| Wife: average number sexual partners in ethnic group | | 1.9 | 0.5 | 1.9 | 0.4 | 1.7 | 0.5 |
| Wife: prevalence of sex before marriage in ethnic group | | 40% | 18% | 42% | 17% | 35% | 20% |
| Husband: average number sexual partners in ethnic group | | 5.7 | 2.4 | 5.5 | 2.2 | 5.1 | 2.4 |
| Husband: prevalence of sex before marriage in ethnic group | | 72% | 18% | 72% | 18% | 66% | 23% |
|  |  |  |  |  |  |  |  |

Table 3. Paternity concern: Results of multilevel multivariate logistic regression control models testing the association between IPPV and IPSV in the past 12 months and independent variables relating to paternity concern hypotheses.

Results show the effect of adding each independent variable separately to the control variables.

Country (n 12) Ethnic groups (n 103) Couples (n 20,610)

|  |  | **IPPV** | | | | **IPSV** | | | |
| --- | --- | --- | --- | --- | --- | --- | --- | --- | --- |
|  |  | OR | 95% CI | | p-value | OR | 95% CI | | p-value |
| **Control variables** |  |  |  |  |  |  |  |  |  |
| Household wealth (Poorest) | Poorer | 1.17 | (1.02 | - 1.32) | 0.020 * | 0.96 | (0.81 | - 1.13) | 0.631 |
|  | Middle | 1.19 | (1.03 | - 1.36) | 0.012 * | 1.01 | (0.85 | - 1.20) | 0.865 |
|  | Richer | 0.97 | (0.83 | - 1.13) | 0.728 | 0.96 | (0.79 | - 1.17) | 0.696 |
|  | Richest | 0.93 | (0.76 | - 1.11) | 0.428 | 0.69 | (0.53 | - 0.88) | 0.004 ** |
| Household residence (Urban) | Rural | 0.84 | (0.75 | - 0.95) | 0.007 ** | 1.00 | (0.85 | - 1.17) | 0.984 |
| Husband’s education (None) | Primary | 1.06 | (0.93 | - 1.21) | 0.366 | 1.03 | (0.85 | - 1.24) | 0.740 |
|  | Secondary | 1.10 | (0.95 | - 1.28) | 0.174 | 0.93 | (0.76 | - 1.15) | 0.552 |
|  | Higher | 0.76 | (0.60 | - 0.96) | 0.024 * | 0.74 | (0.52 | - 1.05) | 0.093 |
| Husband’s religion (Muslim) | Christian | 0.80 | (0.67 | - 0.94) | 0.009 ** | 0.88 | (0.68 | - 1.14) | 0.338 |
|  | Other/none | 0.92 | (0.73 | - 1.15) | 0.470 | 0.91 | (0.65 | - 1.29) | 0.615 |
| Husband drinks alcohol (No) | Yes | 2.66 | (2.42 | - 2.93) | 0.000 *** | 1.49 | (1.31 | - 1.70) | 0.000 *** |
| Husband's age |  | 0.97 | (0.96 | - 0.98) | 0.000 *** | 0.99 | (0.98 | - 1.01) | 0.876 |
| Wife's age |  | 1.00 | (0.99 | - 1.01) | 0.463 | 0.99 | (0.98 | - 1.01) | 0.685 |
| No. IPV justifications husband agrees with |  | 1.08 | (1.05 | - 1.11) | 0.000 *** | 1.05 | (1.00 | - 1.09) | 0.021 * |
| Husband ever paid for sex (No) | Yes | 1.03 | (0.93 | - 1.23) | 0.339 | 0.84 | (0.69 | - 1.01) | 0.177 |
| Childhood exposure to IPV (%) |  | 1.05 | (1.01 | - 1.03) | 0.000 *** | 1.01 | (1.00 | - 1.03) | 0.012 * |
| Wife: More than one marriage (No) | Yes | 1.16 | (1.02 | - 1.32) | 0.017 * | 1.22 | (1.03 | - 1.43) | 0.016 * |
| Husband: More than one marriage (No) | Yes | 1.22 | (1.10 | - 1.34) | 0.000 *** | 1.03 | (0.90 | - 1.18) | 0.571 |
| Sexual IPV (No) | Yes | 7.69 | (6.85 | - 8.64) | 0.000 *** | .. | .. | .. | .. |
| Physical IPV (No) | Yes | .. | .. | .. | .. | 7.81 | (6.92 | - 8.82) | 0.000 *** |
| **Independent variables (individual level)** | |  |  |  |  |  |  |  |  |
| Husband jealous (No) | Yes | 2.59 | (2.35 | - 2.84) | 0.000 *** | 2.24 | (1.97 | - 2.55) | 0.000 *** |
| Husband accuses of infidelity (No) | Yes | 3.35 | (3.04 | - 3.69) | 0.000 *** | 2.28 | (2.02 | - 2.58) | 0.000 *** |
| Husband insists knowing where wife is (No) | Yes | 1.95 | (1.79 | - 2.13) | 0.000 *** | 2.85 | (2.52 | - 3.23) | 0.000 *** |
| Wife: sex before marriage (No) | Yes | 1.14 | (1.04 | - 1.25) | 0.003 ** | 1.06 | (0.94 | - 1.20) | 0.291 |
| Wife: no. sexual partners (one) | Two | 1.23 | (1.10 | - 1.38) | 0.000 *** | 1.03 | (0.88 | - 1.20) | 0.662 |
|  | Three | 1.41 | (1.21 | - 1.65) | 0.000 *** | 1.71 | (1.41 | - 2.07) | 0.000 *** |
|  | Four or more | 1.58 | (1.30 | - 1.91) | 0.000 *** | 1.67 | (1.31 | - 2.13) | 0.000 *** |
| Husband: sex before marriage (No) | Yes | 1.02 | (0.92 | - 1.13) | 0.619 | 1.03 | (0.90 | - 1.18) | 0.065 |
| Husband: no. sexual partners (one) | Two | 1.08 | (0.93 | - 1.26) | 0.286 | 1.14 | (0.94 | - 1.40) | 0.610 |
|  | Three | 1.14 | (0.97 | - 1.33) | 0.105 | 1.09 | (0.88 | - 1.35) | 0.173 |
|  | Four or more | 1.10 | (0.92 | - 1.31) | 0.284 | 1.04 | (0.82 | - 1.32) | 0.426 |
| **Independent variables (ethnic group level)** | |  |  |  |  |  |  |  |  |
| Women: Sex before marriage prevalence % | | 1.00 | (0.99 | - 1.00) | 0.437 | 1.00 | (0.99 | - 1.01) | 0.262 |
| Women: Mean number of lifetime sexual partners | | 1.14 | (0.92 | - 1.41) | 0.203 | 1.10 | (0.81 | - 1.50) | 0.516 |
| Men: Sex before marriage prevalence % | | 1.00 | (1.00 | - 1.01) | 0.058 | 0.99 | (0.98 | - 1.00) | 0.554 |
| Men: Mean number of lifetime sexual partners | | 1.02 | (0.98 | - 1.07) | 0.180 | 0.96 | (0.90 | - 1.03) | 0.347 |
|  |  |  |  |  |  |  |  |  |  |

Notes:

Reference categories for categorical variables are shown in brackets

Table 4. Reproductive conflict: Results of multilevel multivariate logistic regression control models testing the association between IPPV and IPSV in the past 12 months and independent variables relating to reproductive conflict hypotheses.

Results show the effect of adding each independent variable separately to the control variables.

Country (n 12) Ethnic groups (n 103) Couples (n 24,577)

|  |  | **IPPV** | | | | **IPSV** | | | |
| --- | --- | --- | --- | --- | --- | --- | --- | --- | --- |
| **Control variables** |  | OR | 95% CI | | p-value | OR | 95% CI | | p-value |
| Household wealth (Poorest) | Poorer | 1.15 | (1.02 | - 1.29) | 0.017 * | 0.97 | (0.83 | - 1.13) | 0.737 |
|  | Middle | 1.19 | (1.05 | - 1.34) | 0.005 ** | 1.03 | (0.88 | - 1.21) | 0.665 |
|  | Richer | 0.96 | (0.84 | - 1.10) | 0.617 | 0.88 | (0.74 | - 1.06) | 0.194 |
|  | Richest | 0.89 | (0.75 | - 1.05) | 0.194 | 0.67 | (0.53 | - 0.84) | 0.001 ** |
| Household residence (Urban) | Rural | 0.83 | (0.74 | - 0.92) | 0.001 ** | 0.97 | (0.83 | - 1.12) | 0.705 |
| Husband's education (None) | Primary | 1.08 | (0.96 | - 1.21) | 0.199 | 1.10 | (0.93 | - 1.29) | 0.259 |
|  | Secondary | 1.10 | (0.96 | - 1.25) | 0.159 | 0.95 | (0.79 | - 1.14) | 0.606 |
|  | Higher | 0.74 | (0.60 | - 0.92) | 0.008 ** | 0.76 | (0.56 | - 1.05) | 0.101 |
| Husband’s religion (Muslim) | Christian | 0.79 | (0.68 | - 0.92) | 0.003 ** | 0.94 | (0.74 | - 1.19) | 0.628 |
|  | Other/none | 0.89 | (0.73 | - 1.09) | 0.274 | 0.98 | (0.72 | - 1.34) | 0.945 |
| Husband drinks alcohol (No) | Yes | 2.70 | (2.47 | - 2.95) | 0.000 *** | 1.53 | (1.36 | - 1.72) | 0.000 *** |
| No. IPV justifications husband agrees with |  | 1.07 | (1.04 | - 1.10) | 0.000 *** | 1.06 | (1.02 | - 1.10) | 0.001 ** |
| Husband's age |  | 0.98 | (0.97 | - 0.98) | 0.000 *** | 1.00 | (0.99 | - 1.01) | 0.937 |
| Wife's age |  | 1.00 | (0.99 | - 1.01) | 0.396 | 0.99 | (0.98 | - 1.00) | 0.528 |
| Childhood exposure to IPV (%) |  | 1.02 | (1.01 | - 1.03) | 0.000 *** | 1.01 | (1.00 | - 1.02) | 0.052 |
| Sexual IPV (No) | Yes | 7.79 | (7.02 | - 8.64) | 0.000 *** |  |  |  |  |
| Physical IPV (No) | Yes |  |  |  |  | 8.06 | (7.23 | - 9.00) | 0.000 *** |
| **Independent variables: reproductive coercion** | |  |  |  |  |  |  |  |  |
| Husband's fertility desire (Wants no more) | |  |  |  |  |  |  |  |  |
| Wants more within 2 yrs | | 1.00 | (0.89 | - 1.13) | 0.913 | 0.96 | (0.82 | - 1.13) | 0.675 |
| Wants more after 2 yrs | | 0.99 | (0.89 | - 1.10) | 0.877 | 0.93 | (0.81 | - 1.07) | 0.319 |
| Unsure/infertile | | 1.00 | (0.86 | - 1.17) | 0.919 | 0.80 | (0.64 | - 0.99) | 0.048 * |
| Wife's fertility desire (Wants no more) | |  |  |  |  |  |  |  |  |
| Wants more within 2 yrs | | 1.15 | (0.99 | - 1.33) | 0.067 | 1.17 | (0.96 | - 1.43) | 0.107 |
| Wants more after 2 yrs | | 1.01 | (0.88 | - 1.17) | 0.795 | 0.96 | (0.79 | - 1.15) | 0.667 |
| Unsure/infertile | | 1.21 | (1.05 | - 1.39) | 0.007 ** | 0.99 | (0.83 | - 1.19) | 0.984 |
| Fertility desire comparison (Both want no more) | |  |  |  |  |  |  |  |  |
| Disagree: husband wants more/sooner | | 1.13 | (1.03 | - 1.25) | 0.010 * | 1.03 | (0.90 | - 1.17) | 0.657 |
| Disagree: wife wants more/sooner | | 1.10 | (0.98 | - 1.22) | 0.081 | 1.21 | (1.05 | - 1.40) | 0.007 ** |
| Either unsure | | 0.96 | (0.83 | - 1.11) | 0.617 | 0.86 | (0.71 | - 1.05) | 0.153 |
| Wife's no. living children |  | 1.02 | (0.99 | - 1.04) | 0.091 | 1.00 | (0.97 | - 1.04) | 0.628 |
| Husband no. living children | | 1.02 | (1.01 | - 1.08) | 0.001 ** | 1.02 | (0.99 | - 1.04) | 0.105 |
| **Independent variables: paternal disinvestment** | |  |  |  |  |  |  |  |  |
| Living children comparison (Both have the same) | |  |  |  |  |  |  |  |  |
| Wife has more | | 1.10 | (0.96 | - 1.26) | 0.164 | 1.15 | (0.95 | - 1.38) | 0.137 |
| Husband has more | | 1.21 | (1.11 | - 1.33) | 0.000 *** | 1.14 | (1.02 | - 1.28) | 0.020 * |
| Polygamous (No) | Yes | 1.33 | (1.18 | - 1.50) | 0.000 *** | 1.06 | (0.90 | - 1.24) | 0.483 |
| Husband had extramarital sex last 12 m (No) Yes | | 1.33 | (1.17 | - 1.50) | 0.000 *** | 1.08 | (0.91 | - 1.27) | 0.346 |
| Wife's economic independence (Not working) | |  |  |  |  |  |  |  |  |
| No earnings | | 0.90 | (0.81 | - 1.01) | 0.092 | 1.15 | (1.35 | - 1.79) | 0.000 *** |
| Paid in cash | | 1.02 | (0.93 | - 1.13) | 0.567 | 1.66 | (1.01 | - 1.35) | 0.028 * |
| Paid in cash/kind | | 1.08 | (0.94 | - 1.24) | 0.262 | 1.16 | (1.37 | - 2.01) | 0.000 *** |
|  |  |  |  |  |  |  |  |  |  |

Notes:

Reference categories for categorical variables are shown in brackets

Supplementary Table 5. Summary of studies using multivariate logistic regression analysis to examine men’s self-reported IPV behaviour in low and middle income countries

| **Reference** | **Country** | **Sample size** | **IPV type and prevalence** | | **INDIVIDUAL** | | | | | | | | | | | | | | **COUPLE** | | | | | | | **COMMUNITY** | |
| --- | --- | --- | --- | --- | --- | --- | --- | --- | --- | --- | --- | --- | --- | --- | --- | --- | --- | --- | --- | --- | --- | --- | --- | --- | --- | --- | --- |
|  |  |  | Physical | Sexual | Socioeconomic profile | | | | | Childhood experience | | Behaviour/adult experiences | | | | Attitude | Health | |  |  |  |  |  |  |  |  |  |
|  |  |  | L = lifetime  T = past 10 years  Y = past year  C = with current partner | | **Poverty/ economic stress** | Young age at first marriage | Low/high education | In employment | Age (i.e. older man) | Adverse childhood experience e.g. abuse, trauma, neglect | Witnessing parental violence in childhood | Involved in violence or gangs with other men | **Pre/extra marital sex, multiple sexual partners** | Drug or alcohol use / abuse | Transactional sex | Inequitable gender attitudes, IPV justified | STI or HIV+ | PTSD, depression, poor mental health | Relationship conflict, instability | **Conflict due to female infidelity** | **Conflict due to male infidelity** | Marital difference (e.g. in age/education) | Years married | Multiple children | Childlessness | Community norms of IPV | Community violence |
| (Hoffman et al., 1994) | Thailand | 619 | **L** 20 % | .. | **x** | .. | .. | n/s | .. | .. | .. | .. | .. | n/s | .. | .. | .. | n/s | **x** | .. | .. | n/s | n/s | n/s | .. | .. | .. |
| (Martin et al., 1999) | India: 5 districts | 6,156 | 18-45% **L**  (analysis IPPV only) | **L** 4-40% | **x** | **v** | **x** (-) | .. | .. | .. | .. | .. | .. | .. | .. | .. | .. | .. | .. | .. | .. | .. | **v** | **v** | n/s | .. | .. |
| (Martin et al., 2002) | India | 6,902 | 26% **L** | .. | **x** | .. | **x** (-) | .. | n/s | .. | **x** | .. | .. | .. | .. | .. | .. | .. | .. | .. | .. | .. | n/s | n/s | .. | .. | .. |
|  |  |  | .. | 51% **L** | **x** | .. | n/s | .. | n/s | .. | **x** | .. | .. | .. | .. | .. | .. | .. | .. | .. | .. | .. | n/s | n/s | .. | .. | .. |
| (Abrahams et al., 2004a) | South Africa | 1,368 | .. | 15% **T** ^a)^ | .. | .. | n/s | .. | n/s | n/s | n/s | **x** | **x** | **x** | .. | **x** | .. | .. | **x** ^b)^ | .. | .. | .. | .. | .. | .. | .. | .. |
| (Abrahams et al., 2006) | South Africa | 1,378 | 42% T  9% Y | .. | .. | .. | **x** (-) | .. | .. | n/s | n/s | n/s | **x** | **x x** | .. | **x** | .. | .. | **x** | .. | **x** | .. | .. | .. | .. | .. | .. |
| (Dunkle et al., 2006) | South Africa | 1,275 | 22.9% Y |  | .. | .. | .. | .. | .. | .. | .. | **x** | **x** | **x x** | **x** | .. | .. | .. | .. | .. | .. | .. | .. | .. | .. | .. | .. |
|  |  |  |  | 3.6% Y | .. | .. | .. | .. | .. | .. | .. | **x** | **x** | **x** n/s | **x** | .. | .. | .. | .. | .. | .. | .. | .. | .. | .. | .. | .. |
| (Koenig et al., 2006) | India | 4,520 | **25.1% Y** 31.8% L |  | **x** | .. | **x** (-) | .. | .. | .. | **x** | .. | **x** | .. | .. | .. | .. | .. | .. | .. | .. | .. | **x** (+) | .. | **x** | **x** | **x** |
|  |  |  |  | **30.1% Y** 34.1% L | **x** | .. | **x** (+) | .. | .. | .. | **x** | .. | **x** | .. | .. | .. | .. | .. | .. | .. | .. | .. | n/s | .. | **x** | n/s | **x** |
| (Silverman et al., 2007) | Bangladesh ^c)^ | 3,096 | 20.5% Y  (Both: 7.7% ) |  | n/i | n/i | n/i | n/i | n/i | .. | .. | .. | **x x** | .. | .. | .. | **x** | .. | .. | .. | .. | .. | .. | .. | .. | .. | .. |
|  |  |  |  | 9.5% Y | n/i | n/i | n/i | n/i | n/i | .. | .. | .. | **x x** | .. | .. | .. | **x** | .. | .. | .. | .. | .. | .. | .. | .. | .. | .. |
| (Aklimunnessa et al., 2007) | Bangladesh ^c)^ | 3,165 | 68% Y  (Any IPV:72%) | 27% Y | .. | .. | .. | .. | .. | .. | .. | .. | **x** | **x** | .. | .. | **x** | .. | .. | .. | .. | .. | .. | .. | .. | .. | .. |
| (Gupta et al., 2008) | South Africa | 834 | 28% C | .. | n/s |  | n/s | n/s | n/s | **x** | **x** | .. | .. | .. | .. | .. | .. | .. | **x** ^g)^ | .. | .. | .. | .. | **x** ^d)^ | .. | .. | .. |
| (Pulerwitz and Barker, 2008) | Brazil ^e)^ | 223 | 31% C | .. | .. | .. | .. | .. | .. | .. | .. | .. | .. | .. | .. | **x** | .. | .. | .. | .. | .. | .. | .. | .. | .. | .. | .. |
| (Sambisa et al., 2010) | Bangladesh | 1,508 | 55% L  (23% Y) |  | **x** | .. | **x** ^f)^ | n/s | **x** | .. | .. | .. | .. | **x** | .. | **x** | n/s | **x** | .. | .. | .. | .. | .. | .. | .. | .. | .. |
|  |  |  |  | 20% L | **x** | .. | n/s | n/s | n/s | .. | .. | .. | .. | **x** | .. | **x** | **x** | n/s | .. | .. | .. | .. | .. | .. | .. | .. | .. |
| (Townsend et al., 2011) | South Africa | 430 | 36% Y |  | .. | .. | .. | .. | .. | .. | .. | .. | n/s | **x** | .. | .. | **x** | .. | .. | **x** | .. | .. | .. | .. | .. | .. | .. |
|  |  |  |  | 18.9% Y | .. | .. | .. | .. | .. | .. | .. | .. | **x** | ns | .. | .. | **x** | .. | .. | **x** | .. | .. | .. | .. | .. | .. | .. |
| (Fulu et al., 2013) | 9 countries Asia & Pacific | 10,178 | 6 - 45% L  (Both: 4-41%) | .. | **x** | .. | **x** (-) | .. | .. | **x** | .. | **x** | .. | **x** | .. | .. | .. | **x** | .. | .. | .. | .. | .. | .. | .. | .. | **x** |
|  |  |  |  | 3 – 22% L | n/s | .. | n/s | .. | .. | **x** | .. | n/s | .. | **x** | .. | .. | .. | n/s | .. | .. | .. | .. | .. | .. | .. | .. | n/s |
| (Fleming et al., 2015) | 8 countries (IMAGES) | 7,806 | 31% (range 17-45%) L | .. | **x** | .. | n/s | n/s | **x** | .. | **x** | **x** | .. | .. | .. | **x** | .. | .. | .. | .. | .. | .. | .. | .. | .. | .. |  |
| (Barker et al., 2015) | 8 countries | 8,000 | 17-39% L |  | **x** | .. | **x** (-) | .. | **x** (-) | .. | **x** | .. | .. | **x** | .. | **x** | .. | .. | .. | .. | .. | .. | .. | .. | .. | **x** | .. |
|  |  |  |  | 25% (range 6-29%) L | n/s | .. | n/s | .. | n/s | .. | **x** | .. | .. | **x** | .. | **x** | .. | .. | .. | .. | .. | .. | .. | .. | .. | n/s | .. |
| (Fonseka et al., 2015) ** | Sri Lanka | 1,252 | 21.9% L  Any: 49.3% |  | n/i | n/i | n/i | n/i | n/i | **x x** | **x** | .. | .. | .. | .. | .. | .. | .. | .. | .. | .. | .. | .. | .. | .. | .. | .. |
|  |  |  |  | 13.7% L | n/i | n/i | n/i | n/i | n/i | **x x** | n/s | .. | .. | .. | .. | .. | .. | .. | .. | .. | .. | .. | .. | .. | .. | .. | .. |
| (Machisa et al., 2016) | South Africa | 416 | Any IPV  44% L |  | .. | .. | .. | **x** | .. | **x** | .. | .. | .. | .. | .. | .. | .. | **x** | .. | .. | .. | .. | .. | .. | .. | .. | .. |
| (Peitzmeier et al., 2016) | USA, India, China, South Africa | 2,013 | any IPV  9-40% Y |  | .. | .. | **v** | **v** | **v** | **x** | .. | **x** | .. | **x** | .. | .. | .. | **x** | .. | .. | .. | .. | .. | .. | .. | .. | .. |
| (VanderEnde et al., 2016) | Malawi | 450 | 9% L |  | .. | .. | .. | .. | .. | **x** ^h)^ | .. | n/s | .. | .. | .. | .. | .. | .. | .. | .. | .. | .. | .. | .. | .. | .. | n/s |
|  |  |  |  | 24% L | .. | .. | .. | .. | .. | **x** ^i)^ | .. | **x** | .. | .. | .. | .. | .. | .. | .. | .. | .. | .. | .. | .. | .. | .. | n/s |
| (Yount et al., 2016) | Vietnam | 522 | 28% L  any IPV ^j)^ | 0.2% | n/s | .. | .. | .. | n/s | **x** | **x** | .. | .. | .. | .. | **x** | .. | .. | .. | .. | .. | **x** | .. | n/s | .. | **x** | .. |
| (Akhter and Wilson, 2016) | Bangladesh | 3,339 | **74% L**  (37% Y) | .. | **x** | .. | **x** (-) | .. | **x** | .. | .. | .. | .. | n/s | .. | n/s | .. | .. | .. | n/s | **x** | .. | .. | .. | .. | .. | .. |
| (Teitelman et al., 2017) | South Africa | 871 | Any IPV 21.8% Y |  | .. | .. | n/s | **x** | .. | **x** | .. | .. | .. | **x** | .. | .. | .. | .. | **x** | .. | .. | **x** | .. | .. | .. | .. | .. |
| (Gilchrist et al., 2017) | Brazil/England | 281/223 | 46.4% L | 11.6% L | .. | .. | .. | .. | .. | **x** | .. | **x** | .. | **x** | .. | .. | .. | **x** | .. | .. | .. | .. | .. | .. | .. | .. | .. |
| (Yount et al., 2018) | Bangladesh | 1,508 | 50% L |  | **x** | .. | **x** (-) | .. | **x** | **x** | **x** | .. | .. | .. | .. | **x** | .. | .. | .. | .. | .. | **x** | .. | **x** | .. | **x** | .. |
| (Chirwa et al., 2018) ** | Ghana | 2,126 | (27.7% L)  25.0% Y | (27.8% L)  16.6% Y | n/s | .. | .. | n/s | n/s | **x** | **x** | .. | **x** | **x** | **x** | **x** | .. | n/s | n/s | .. | .. | n/s | .. | .. | .. | .. | .. |
| (Machisa and Shamu, 2018) | Zimbabwe | 2,838 | 56% L | 31% | .. | .. | .. | .. | **x** | **x** | .. | .. | **x** | **x** | .. | **x** | .. | **x** | .. | .. | .. | .. | .. | .. | .. | .. | .. |

| Notes  ^a)^ 80.9% of men reporting sexual IPV also reported physical IPV during the same period.  ^b)^ Specifically conflict over sexual refusal and conflict where men perceived their authority to be undermined  ^c)^ Both these studies used Bangladesh DHS datasets from 2004 survey – it is unclear how they have such different estimates of IPV prevalence.  ^d)^ Having 1-2 children (compared to no children, or 3+ children) was associated with men’s use of IPPV  ^e)^ Factor analysis not logistic regression  ^f)^ IPPV significantly associated with primary education, rather than no education or secondary education  ^g)^ Being separated or divorced associated with IPPV  ^h)^ Physical and emotional childhood abuse is significantly associated with IPPV  ^i)^ Sexual and emotional childhood abuse is significantly associated with IPSV  ^j)^ Includes physical, psychological and sexual IPV | ** study captured frequency of IPV, not just binary response  **x** = variable significant in multivariate logistic regression  **v** = significance of variable varied in multi-country study  n/s = variable not significant in multivariate logistic regression  n/I = variable included in model but no information on significance provided  .. = variable not included in the multivariate logistic regression model |
| --- | --- |

ABRAHAMS, N., JEWKES, R., HOFFMAN, M. & LAUBSCHER, R. 2004. Sexual violence against intimate partners in Cape Town: prevalence and risk factors reported by men. *Bulletin of The World Health Organization,* 82**,** 330-337.

ABRAHAMS, N., JEWKES, R., LAUBSCHER, R. & HOFFMAN, M. 2006. Intimate partner violence: Prevalence and risk factors for men in Cape Town, South Africa. *Violence and victims,* 21**,** 247-264.

AKHTER, R. & WILSON, J. K. 2016. Using an Ecological Framework to Understand Men’s Reasons for Spousal Abuse: An Investigation of the Bangladesh Demographic and Health Survey 2007. *Journal of Family Violence,* 31**,** 27-38.

AKLIMUNNESSA, K., KHAN, M. M. H., KABIR, M. & MORI, M. 2007. Prevalence and correlates of domestic violence by husbands against wives in Bangladesh: evidence from a national survey. *The Journal of Men's Health & Gender,* 4**,** 52-63.

BARKER, G., CONTRERAS, J., HEILMAN, B., SINGH, A., VERMA, R. & NASCIMENTO, M. 2015. *Evolving men: initial results from the international men and gender equality survey (IMAGES)*. Washington DC:ICRW

DUNKLE, K. L., JEWKES, R. K., NDUNA, M., LEVIN, J., JAMA, N., KHUZWAYO, N., KOSS, M. P. & DUVVURY, N. 2006. Perpetration of partner violence and HIV risk behaviour among young men in the rural Eastern Cape, South Africa. *AIDS,* 20**,** 2107-2114.

FLEMING, P. J., MCCLEARY-SILLS, J., MORTON, M., LEVTOV, R., HEILMAN, B. & BARKER, G. 2015. Risk Factors for Men’s Lifetime Perpetration of Physical Violence against Intimate Partners: Results from the International Men and Gender Equality Survey (IMAGES) in Eight Countries. *PLOS ONE,* 10**,** e0118639.

FONSEKA, R. W., MINNIS, A. M. & GOMEZ, A. M. 2015. Impact of Adverse Childhood Experiences on Intimate Partner Violence Perpetration among Sri Lankan Men. *Plos One,* 10.

FULU, E., JEWKES, R., ROSELLI, T., GARCIA-MORENO, C. & ME, U. N. M. C. S. 2013. Prevalence of and factors associated with male perpetration of intimate partner violence: findings from the UN Multi-country Cross-sectional Study on Men and Violence in Asia and the Pacific. *Lancet Global Health,* 1**,** E187-E207.

GILCHRIST, G., RADCLIFFE, P., NOTO, A. R. & PIRES LUCAS D'OLIVEIRA, A. F. 2017. The prevalence and factors associated with ever perpetrating intimate partner violence by men receiving substance use treatment in Brazil and England: A cross-cultural comparison. *Drug and Alcohol Review,* 36**,** 34-51.

GUPTA, J., SILVERMAN, J. G., HEMENWAY, D., ACEVEDO-GARCIA, D., STEIN, D. J. & WILLIAMS, D. R. 2008. Physical violence against intimate partners and related exposures to violence among South African men. *Canadian Medical Association Journal,* 179**,** 535.

HOFFMAN, K. L., DEMO, D. H. & EDWARDS, J. N. 1994. Physical Wife Abuse in a Non-Western Society: An Integrated Theoretical Approach. *Journal of Marriage and Family,* 56**,** 131-146.

KOENIG, M. A., STEPHENSON, R., AHMED, S., JEJEEBHOY, S. J. & CAMPBELL, J. 2006. Individual and Contextual Determinants of Domestic Violence in North India. *American Journal of Public Health,* 96**,** 132-138.

MACHISA, M. T., CHRISTOFIDES, N. & JEWKES, R. 2016. Structural Pathways between Child Abuse, Poor Mental Health Outcomes and Male-Perpetrated Intimate Partner Violence (IPV). *Plos One,* 11.

MACHISA, M. & SHAMU, S. 2018. Mental ill health and factors associated with men's use of intimate partner violence in Zimbabwe. *Bmc Public Health,* 18.

MARTIN, S. L., TSUI, A. O., MAITRA, K. & MARINSHAW, R. 1999. Domestic Violence in Northern India. *American Journal of Epidemiology,* 150**,** 417-426.

MARTIN, S. L., MORACCO, K. E., GARRO, J., TSUI, A. O., KUPPER, L. L., CHASE, J. L. & CAMPBELL, J. C. 2002. Domestic violence across generations: findings from northern India. *International Journal of Epidemiology,* 31**,** 560-572.

PEITZMEIER, S. M., KAGESTEN, A., ACHARYA, R., CHENG, Y., DELANY-MORETLWE, S., OLUMIDE, A., BLUM, R. W., SONENSTEIN, F. & DECKER, M. R. 2016. Intimate Partner Violence Perpetration Among Adolescent Males in Disadvantaged Neighborhoods Globally. *Journal of Adolescent Health,* 59**,** 696-702.

PULERWITZ, J. & BARKER, G. 2008. Measuring Attitudes toward Gender Norms among Young Men in Brazil:Development and Psychometric Evaluation of the GEM Scale. *Men and Masculinities,* 10**,** 322-338.

SAMBISA, W., ANGELES, G., LANCE, P. M., NAVED, R. T., CURTIS, S., XE & N, L. 2010. Physical and Sexual Abuse of Wives in Urban Bangladesh: Husbands' Reports. *Studies in Family Planning,* 41**,** 165-178.

SILVERMAN, J. G., DECKER, M. R., KAPUR, N. A., GUPTA, J. & RAJ, A. 2007. Violence against wives, sexual risk and sexually transmitted infection among Bangladeshi men. *Sexually Transmitted Infections,* 83**,** 211-215.

TOWNSEND, L., JEWKES, R., MATHEWS, C., JOHNSTON, L. G., FLISHER, A. J., ZEMBE, Y. & CHOPRA, M. 2011. HIV Risk Behaviours and their Relationship to Intimate Partner Violence (IPV) Among Men Who Have Multiple Female Sexual Partners in Cape Town, South Africa. *AIDS and Behavior,* 15**,** 132-141.

TEITELMAN, A. M., BELLAMY, S. L., JEMMOTT, J. B., III, ICARD, L., O'LEARY, A., ALI, S., NGWANE, Z. & MAKIWANE, M. 2017. Childhood Sexual Abuse and Sociodemographic Factors Prospectively Associated with Intimate Partner Violence Perpetration Among South African Heterosexual Men. *Annals of Behavioral Medicine,* 51**,** 170-178.

VANDERENDE, K., MERCY, J., SHAWA, M., KALANDA, M., HAMELA, J., MAKSUD, N., ROSS, B., GUPTA, S., WADONDA-KABONDO, N. & HILLIS, S. 2016. Violent experiences in childhood are associated with men's perpetration of intimate partner violence as a young adult: a multistage cluster survey in Malawi. *Annals of Epidemiology,* 26**,** 723-728.

YOUNT, K. M., JAMES-HAWKINS, L., CHEONG, Y. F. & NAVED, R. T. 2018. Men's Perpetration of Partner Violence in Bangladesh: Community Gender Norms and Violence in Childhood. *Psychology of Men & Masculinity,* 19**,** 117-130.

YOUNT, K. M., HIGGINS, E. M., VANDERENDE, K. E., KRAUSE, K. H., MINH, T. H., SCHULER, S. R. & ANH, H. T. 2016. Men's Perpetration of Intimate Partner Violence in Vietnam: Gendered Social Learning and the Challenges of Masculinity. *Men and Masculinities,* 19**,** 64-84.
